# The antibiotic resistome and microbiota landscape of refugees from Syria, Iraq and Afghanistan in Germany

**DOI:** 10.1186/s40168-018-0414-7

**Published:** 2018-02-20

**Authors:** Robert Häsler, Christian Kautz, Ateequr Rehman, Rainer Podschun, Volker Gassling, Pius Brzoska, Jon Sherlock, Jan-Thorsten Gräsner, Gesine Hoppenstedt, Sabine Schubert, Astrid Ferlinz, Wolfgang Lieb, Matthias Laudes, Femke-Anouska Heinsen, Jens Scholz, Dag Harmsen, Andre Franke, Swantje Eisend, Thomas Kunze, Helmut Fickenscher, Stephan Ott, Philip Rosenstiel, Stefan Schreiber

**Affiliations:** 1 Institute of Clinical Molecular Biology, University Hospital Schleswig-Holstein, Christian Albrecht University of Kiel, Campus Kiel, Rosalind-Franklin-Straße 12, 24105 Kiel, Germany; 2 Pharmaceutical Institute, Department of Clinical Pharmacy, Christian Albrecht University of Kiel, Kiel, Germany; 3 Institute of Infection Medicine, Christian Albrecht University of Kiel, Kiel, Germany; 4 Department of Oral and Maxillofacial Surgery, University Hospital of Schleswig-Holstein, Kiel, Germany; 5 Thermo Fisher Scientific, San Francisco, CA USA; 6 Department of Anaesthesia and Intensive Care Medicine, University Hospital of Schleswig-Holstein, Kiel, Germany; 7 Thermo Fisher Scientific, Life Technologies GmbH, Darmstadt, Germany; 8 POPGEN Biobank and Institute of Epidemiology, Christian Albrecht University of Kiel, Kiel, Germany; 9 Department of Periodontology and Restorative Dentistry, University Hospital Münster, Münster, Germany; 10 Department of Internal Medicine, University Hospital of Schleswig-Holstein, Campus Kiel, Rosalind-Franklin-Straße 12, 24105 Kiel, Germany

**Keywords:** Antibiotic resistance, Resistome, Human, Refugees

## Abstract

**Background:**

Multidrug-resistant bacteria represent a substantial global burden for human health, potentially fuelled by migration waves: in 2015, 476,649 refugees applied for asylum in Germany mostly as a result of the Syrian crisis. In Arabic countries, multiresistant bacteria cause significant problems for healthcare systems. Currently, no data exist describing antibiotic resistances in healthy refugees. Here, we assess the microbial landscape and presence of antibiotic resistance genes (ARGs) in refugees and German controls. To achieve this, a systematic study was conducted in 500 consecutive refugees, mainly from Syria, Iraq, and Afghanistan and 100 German controls. Stool samples were subjected to PCR-based quantification of 42 most relevant ARGs, 16S ribosomal RNA gene sequencing-based microbiota analysis, and culture-based validation of multidrug-resistant microorganisms.

**Results:**

The fecal microbiota of refugees is substantially different from that of resident Germans. Three categories of resistance profiles were found: (i) ARGs independent of geographic origin of individuals comprising BIL/LAT/CMA, ErmB, and mefE; (ii) vanB with a high prevalence in Germany; and (iii) ARGs showing substantially increased prevalences in refugees comprising CTX-M group 1, SHV, vanC1, OXA-1, and QnrB. The majority of refugees carried five or more ARGs while the majority of German controls carried three or less ARGs, although the observed ARGs occurred independent of signatures of potential pathogens.

**Conclusions:**

Our results, for the first time, assess antibiotic resistance genes in refugees and demonstrate a substantially increased prevalence for most resistances compared to German controls. The antibiotic resistome in refugees may thus require particular attention in the healthcare system of host countries.

**Electronic supplementary material:**

The online version of this article (10.1186/s40168-018-0414-7) contains supplementary material, which is available to authorized users.

## Background

Multidrug-resistant organisms (MDROs) represent one of the most serious threats for human health in the twenty-first century as highlighted by the Word Health Organization [1] and as outlined in the 2016 declaration of the United Nations [2]. Methicillin-resistant *Staphylococcus aureus* (MRSA), vancomycin-resistant enterococci (VRE), and multidrug-resistant Gram-negative bacteria resistant to extended-spectrum β-lactams (ESBL) and/or carbapenems (CRE) are among the most concerning MDROs worldwide and cause a multitude of hospital- and community-acquired infections with limited treatment options. Infections caused by these pathogens are associated with increased mortality, duration of hospital stay, and hospital costs with large economic impact on public health systems [3]. It is estimated that 25,000 patients die from infections with MDROs each year in Europe, resulting in healthcare-related costs to society of at least EUR 1.5 billon [4]. Without appropriate measures, the worldwide number of deaths attributable to such antimicrobial resistances may increase from 700,000 to 10 million by 2050 and the World Health Organization’s prophecy of a post-antibiotic era may turn into reality soon [5].

Prescription and use of antibiotics in medicine and agriculture result in regional differences in prevalences of antibiotic resistances, as observed for differences between Northern and Southern European countries, as well as between Europe and the Middle East or Asia [6]. At the same time, Europe is currently facing substantial refugee movements: in 2015, 476,649 refugees applied for asylum in Germany as a result of the civil wars in Syria, Iraq, and Afghanistan while in 2016, already 657,855 applications were received until September [7]. The UNHCR estimates the total number of Syrian refugees within the last 5 years to be more than 4.2 million [8]. With high prevalences of MDROs, these refugees might represent a reservoir or vehicle for MDROs when migrating to other countries. The quality and quantity of these resistances remain entirely unmonitored, with unknown consequences for the public health system.

Few studies address the issue of MDRO prevalences in Southern and Eastern Europe. Data on MDRO colonialization and prevalence in refugees is very limited, mostly originating from hospitalized individuals [9], which are at higher risk to carry antibiotic resistances and, thus, do not allow to draw conclusions about a mostly healthy refugee population. Moreover, the available data were generated, employing culture-based methods. Consequently, a relevant part of the resistome remains hidden, as most of the bacteria colonizing the human body cannot be cultivated and may serve themselves as a reservoir for transferable antibiotic resistance genes [10]. German authorities recommend preventive isolation and MDRO screening of refugees when admitted to hospital care [11]. However, recommendations have not been made for MDRO screening during the initial registration procedure, which only includes a brief medical assessment of refugees.

The major objectives of this study were to overcome the current lack of information on the antibiotic resistome in refugees by (i) quantifying major antibiotic resistance genes in stool samples originating from a consecutive examination of refugees from Syria, Afghanistan, Iraq, and neighboring countries and (ii) comparing the obtained data to a random control group of healthy German individuals. Employing microfluidic PCR, next-generation sequencing, and culture-based methods, these observations will, for the first time, enable a quantitative comparison of antibiotic resistance gene prevalences in refugees and German individuals. The findings should raise the attention for antibiotic resistances in general and for antibiotic resistances in refugees in particular and lead to further systematic examinations of the admixture of the refugee resistome into the microbiota of the host country’s population.

## Methods

### Recruitment of participants and sample collection

**Fig. 1:**
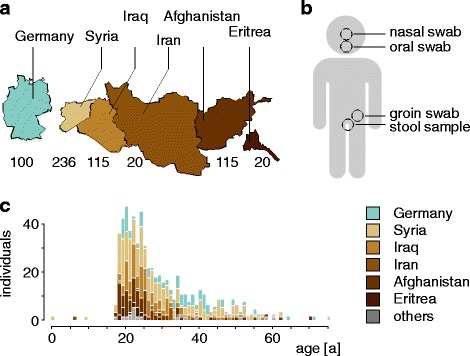
Cohort composition. **a** The number of individuals included in the study from different countries; countries with less than 20 individuals participating are not shown. **b** Different body sites sampled as part of the study. **c** Age distribution of the individuals included in the study, color coded by country. For better visualization, values were binned with 2 years per bin

Refugees participating in this study were recruited during their initial registration for asylum seekers in Neumünster, Germany, in November 2015. In addition to their routine medical examination, samples from 506 sequential, unselected refugees with reported health status were obtained. Registration and sampling was conducted within 7 days after arrival of the refugees in Germany. The sampling procedure included a swab sample from each individual (nasal, oral, and groin) and, if available, a stool sample, routinely collected for the detection of intestinal pathogens. In parallel, epidemiological data was collected, followed by data anonymization. The control group of 100 German individuals comprised age- and gender-matched samples from the population representative PopGen Biobank [12] (FoCus Cohort). For an overview on sampling sites and individuals recruited, see Fig. 1 and Table 1. All participants gave informed consent, and the study protocol was approved by the local ethical committee (D537/15; D501/14).

**Table 1:** Participants overview

Origin (country)	Number of individuals	Gender (f/m)	Age (median; range)
Germany	100	14/86	33; 19–71
Syria	236	33/203	26; 0–75
Iraq	115	13/102	25; 18–57
Afghanistan	95	13/82	23; 17–70
Eritrea	20	1/19	23; 18–43
Iran	20	4/16	32; 6–49
Others^1^	20	7/13	23; 17–60
Total	606	85/521	25; 0–75

^1^Others: refugees from Albania, Armenia, Chechnya, India, Kosovo, Lebanon, Somalia, Turkey, and Yemen

All stool samples originating from refugees were delivered to the microbiology laboratory of the University Medical Centre Schleswig-Holstein, Kiel, within 24 h of collection for routine intestinal pathogen screening. The remaining parts of the samples were stored at − 80 °C until DNA extraction was carried out. Stool samples of German healthy controls were shipped on dried ice from PopGen 2.0 Network (P2N) Biobank and stored at − 80 °C.

All swab tips from one individual were placed together in one sterile tube (Greiner Bio-One, Frickenhausen, Germany), containing 2 ml of sterile sodium chloride 0.85% solution. The swab specimen was stored at 4 °C and further processed up to 14 h.

For further details on processing of swab and stool samples, please refer to Additional file 1: supplemental methods.

### Culture-based detection of MDROs

For the isolation of MDROs, 100 μl of undiluted swab suspension was plated onto the selective media agar plates; for media details, please refer to Additional file 1: Table S1. All resulting colonies were identified on species level by matrix-assisted laser desorption ionization time-of-flight mass spectrometry (Bruker, Bremen, Germany). The respective resistograms were determined using the semi-automated VITEK 2 system (bioMérieux). MRSA isolates were further confirmed by testing for the clumping factor and protein A (Pastorex Staph Plus; Bio-Rad, Munich, Germany) and for the penicillin-binding protein 2a (PBP2a Culture Colony; Alere, Cologne, Germany). The microbiological procedures were performed according to DIN ISO EN 15189/2014 and to the EUCAST guidelines.

### DNA extraction from stool and swab samples

Swab-derived sample suspensions were subjected to a rapid heat lysis protocol, without further purification of the sample. Briefly, this procedure consists of a centrifugation, followed by denaturation in lysis buffer, a heat lysis step (11 min at 99 °C), and stored until further processing at − 80 °C. Stool samples of approximately 0.2 g were extracted, employing the Mo Bio PowerSoil^®^ DNA Isolation Kit (Mo Bio Laboratories, Inc., Carlsbad, USA), and stored until further processing at − 80 °C. For further details, please refer to Additional file 1: Supplemental material.

### Selection of antibiotic resistance genes to be included in the test system employed

Individual bacterial antibiotic resistance genes were included in the setup by an expert selection of the board of the Institute of Infection Medicine, University Clinic of Schleswig-Holstein, while taking the literature on antibiotic resistance genes in the refugee’s countries into account [6, 9, 11, 13–15]. In addition to the 42 resistance genes, three species-specific genes and two endogenous controls were included (see Table 2). Further details (assay IDs, detection limits, and corresponding context sequences) are listed in Additional file 1: Table S2.

**Table 2:** Selected target genes

Category	Target
ampC resistances	ACC; ACT/MIR; ampC; BIL/LAT/CMY; DHA; FOX; MOX/CMY
Carbapenem resistance	IMP-1 group; IMP-16; IMP-2 group; IMP-7; KPC; NDM; OXA-23; OXA-40; OXA-48; OXA-58; OXA-72; VIM
Extended-spectrum beta-lactamase	CTX-M group 1; CTX-M group 2; CTX-M group 8/25; CTX-M group 9; GES; OXA-1; PER-1; PER-2; SHV; TEM; VEB
Macrolide resistance	ErmA; ErmB, mefE
Quinolone and fluoroquinolone resistance	QnrA; QnrB
Vancomycin resistance	vanA1; vanA2; vanB; vanC1; vanC2-C3-1; vanC2-C3-2
Endogenous control	16S (bacterial); GAPDH (human)
Inhibition control, exogenous	exoIPC
Species-specific genes	ACICU_00593 (*Acinetobacter baumannii*); femA SA (*Staphylococcus aureus*); femA SE (*Staphylococcus epidermidis*)

### Detection of antibiotic resistance genes via microfluidic real-time PCR

To detect the 42 of the most common resistance determinants in MRSA, VRE, ESBL, and CRE, a microfluidic real-time PCR system, based on the TaqMan platform (Thermo Fisher, USA), was employed according to the manufacturer’s guidelines and carried out on a ViiA 7 system (Thermo Fisher, USA), supplemented by endogenous and exogenous control assays. Briefly, 54.5 μl of 2× TaqMan^®^ Fast Universal Master Mix (Thermo Fisher, USA) was added to 54.5 μl of lysed swab suspensions and 16.4 μl of stool sample extracts, respectively. Each reaction mix was spiked with 1.0 μl 20× exogenous internal positive control DNA and subsequently loaded on the microfluidic card following the manufacturer’s guidelines. Thermocycling conditions employed were 95 °C for 20 s, followed by 45 cycles of 95 °C for 1 s and 60 °C for 20 s. Real-time PCR data was analyzed with ViiA™ 7 RUO software based on the delta-delta CT method as previously described [16]. Initial performance tests to determine specificity and sensitivity were carried out using bacterial reference samples. All assays with corresponding detection limits are listed in Additional file 1: Table S2. Quantification of antibiotic resistance genes was carried out based on a standard curve derived from dilutions of these reference strains (Additional file 1: Table S3). The system was further validated using 73 clinical samples (Additional file 1: Table S4) with positive MDRO content. For further details, please refer to Additional file 1: Supplemental material.

### Microbiota profiling using 16S ribosomal RNA gene sequencing

In order to investigate microbial communities in feces or swab samples, the 16S ribosomal RNA (rRNA) gene variable regions V3–V4 were amplified using dual-barcoded specific primers [17]. Libraries were generated by pooling an equal amount of barcoded amplicons and sequenced on an Illumina MiSeq platform (Illumina; San Diego, CA, USA). Obtained paired reads were processed using mothur as described [18]. After quality control, sequences were mapped to the taxonomical hierarchy using mothur-curated greengene reference training sets (version 13_8_99) with an 80% confidence threshold and binned in label 1 phylotypes corresponding to genus/species. Diversity index as well as indicator analysis was performed in mothur. For further details on microbiota profiling, including the classification procedure for signatures of potential human pathogens (Additional file 1: Table S5), please refer to Additional file 1: Supplemental material.

## Results

### Prevalences of antibiotic resistance genes in stool samples from refugees and German control individuals

A primary finding of the TaqMan-based quantification of antibiotic resistance genes was that mefA and ermB genes were present in all but one refugee stool sample (prevalence = 99.7%). High prevalence rates in refugees were observed for beta-lactamase genes, including bla_TEM_ (88.1%), bla_CTX-M group 1_ (43.6%), bla_SHV_ (35.0%), bla_BIL/LAT/CMY_ (23.3%), bla_OXA-1_ (19.4%), ampC (15.6%), the quinolone resistance determinant qnrB (28.9%), as well as the glycopeptide resistance gene variant vanC1 (15.3%).

**Fig. 2:**
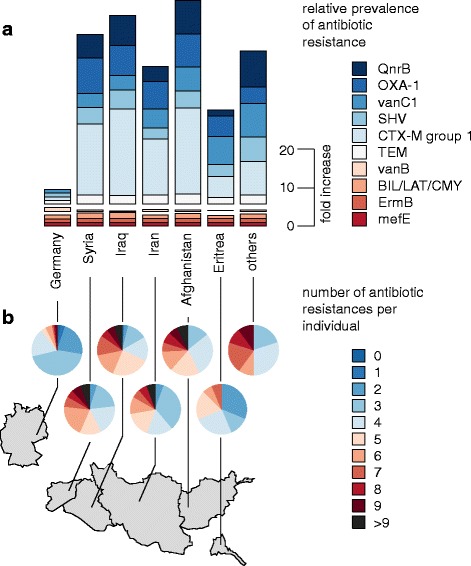
Antibiotic resistances in stool samples from refugees and German control individuals. **a** Prevalences; bar heights represent prevalences relative to German control individuals, color coded by resistance gene. Genes were grouped in three categories: bottom group (BIL/LAT/CMY, ErmB, and mefE), present in similar amounts in refugees and German control individuals; middle group (vanB), present in higher amount in German controls; and upper group (QnrB, OXA-1, vanC1, SHV, CTX-M group 1, TEM), present in higher amounts in refugees, while two resistance genes (QnrB and OXA-1) were not found in German control individuals and scaled separately for visualization purposes. **b** The number of antibiotic resistance genes observed per individual, illustrated by the proportion of individuals per nation with a given number of different resistances. Others: refugees from Albania, Armenia, Chechnya, India, Kosovo, Lebanon, Somalia, Turkey, and Yemen

In German controls, mefE, ermB, TEM, SHV, BIL/LAT/CMY, ampC, DHA, ACC, vanB, and vanC2-C3-2 were the most common genotypes. OXA-1 and qnrB, which were not detected in German controls, were found in refugees of all origins (Fig. 2a; please refer to Additional file 1: Table S6 for a list of all prevalences observed and to supporting materials 02 and 03 for copy numbers/genome equivalents of ARGs in each sample). The majority of all German participants carried three or less antibiotic resistance genes, while the majority of refugees from Syria, Iraq, and Afghanistan carried five or more antibiotic resistance genes (Fig. 2b). A connection between age and the number of antibiotic resistance genes detected could not be observed: Spearman’s rho values for the correlation between the number of resistances and the age ranged from − 0.20 to 0.22 (Germany, 0.14; Syria, 0.22; Iraq, 0.17; Afghanistan, 0.20).

### Prevalences of signatures of potential pathogens in stool samples from refugees and German control individuals

**Fig. 3:**
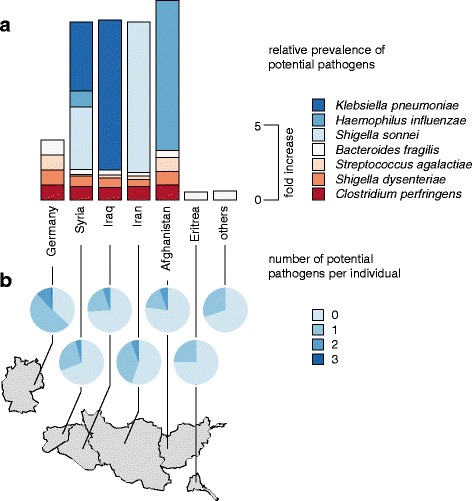
Signatures of potential pathogens in stool samples from refugees and German control individuals. **a** Prevalence of 16S rRNA gene fragments indicative of potential pathogens in refugees and German control individuals. Bar heights represent prevalences relative to German control individuals, color coded by potential pathogen. Potential pathogens, which were not found in German control individuals (*Klebsiella pneumoniae*, *Haemophilus influenzae*, *Shigella sonnei*), were scaled separately for visualization purposes. **b** The number of signatures of potential pathogens observed per individual illustrated by the proportion of individuals per nation with a given number of different potential pathogens. Others: refugees from Albania, Armenia, Chechnya, India, Kosovo, Lebanon, Somalia, Turkey, and Yemen

*Klebsiella pneumonia*, *Haemophilus influenzae*, and *Shigella sonnei*, while present in eight refugees, could not be found in any German sample (Fig. 3a) based on 16S rRNA gene sequencing (see supporting materials 04 and 05 for quantitative 16S rRNA gene sequencing data for each individual). Several other potential pathogenic taxa were identified in refugees and German individuals. Occurrences of signatures of potential pathogens did not show any correlation to antibiotic resistance genes observed. For further details on these results, please refer to Additional file 1: Table S7. The majority of German individuals carried one or more such species, while in all refugee groups, the majority of individuals did not carry any signatures of potential pathogens (Fig. 3b). A connection between age and the number of signatures of potential pathogens detected could not be observed: Spearman’s rho values for the correlation between the number of potential pathogens and the age ranged from − 0.04 to 0.18 (Germany, − 0.04; Syria, 0.18; Iraq, 0.17; Afghanistan, 0.11).

Comparison of prevalence data and estimated copy numbers for antibiotic resistance genes showed that gene copy numbers were relatively stable across different countries, yet the prevalences oftentimes differed substantially (Additional file 1: Figure S1).

### Regional microbiota differences based on 16S rRNA gene sequence analysis

**Fig. 4:**
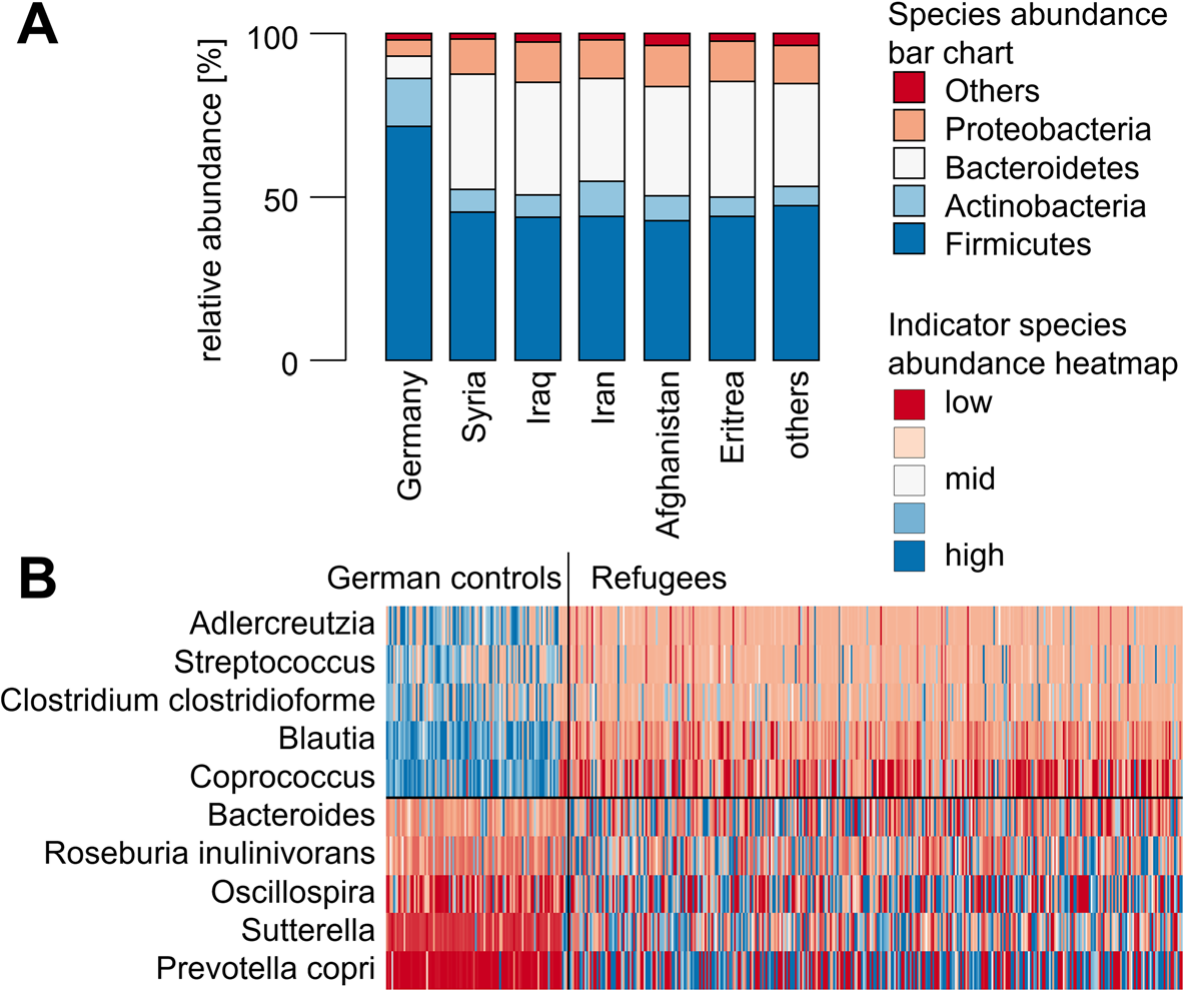
Stool microbiota composition differences between refugees and German control individuals. **a** Relative abundances of major bacterial phyla in German control individuals and refugees. Fecal bacterial profiles were generated by 16S rRNA gene amplicon sequencing. **b** Heatmap of top 10 selected indicator phylotypes, which are more abundant in German control individuals (upper half) and in refugees (lower half), color coded by abundance, which was z-score normalized for better visualization

16S rRNA gene sequences were dominated by known gut bacterial phyla Firmicutes, Bacteroidetes, Proteobacteria, and Actinobacteria (Fig. 4a). The gut microbiota of German control individuals contained significantly higher abundances of Firmicutes and Actinobacteria, whereas Bacteroidetes and Proteobacteria were significantly more abundant in refugee groups (for *p* values, refer to Additional file 1: Table S8). However, alpha diversity indices in all populations were comparable (Additional file 1: Figure S2). A subsequent correlation analysis between microbial taxa and antibiotic resistance genes showed that (i) selected bacterial taxa exhibiting a negative correlation to ARGs in German individuals show partially opposite correlations in refugees (Additional file 1: Figure S3A) and (ii) the distribution of correlations of all bacterial taxa was shifted towards negative values in German individuals, when compared to refugees on the level of individual ARGs (Additional file 1: Figure S3B).

In a next step, all refugee profiles were combined and compared to German control individuals, aiming to identify indicator bacterial phylotypes. This analysis identified 68 and 67 indicator phylotypes (*p* < 0.05) in German and refugee populations, respectively (Fig. 4b). Top five indicator phylotypes in German controls were from Firmicutes (*Blautia*, *Streptococcus*, *Coprococcus*, *Clostridium clostridioforme*) and Actinobacteria (*Adlercreutzia*), whereas in refugees, the top indicators were from the phyla Proteobacteria (*Sutterella*) and from Bacteroidetes (*Prevotella copri*, *Bacteroides*) and Firmicutes (*Oscillospira*, *Roseburia*).

**Fig. 5:**
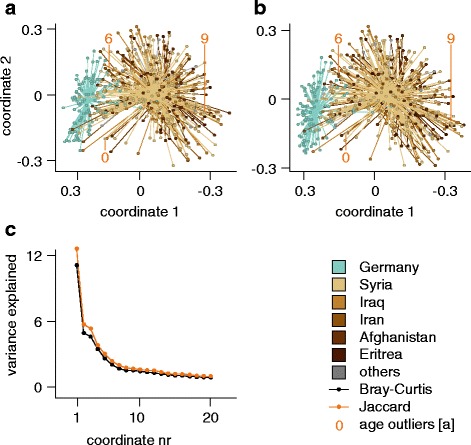
Regional microbiota differences based on stool 16S rRNA gene analysis. Principal coordinate analysis (PCoA) based on the Bray-Curtis index (**a**) and on the Jaccard index (**b**), color coded by origin. Individuals below 10 years of age are labeled specifically. **c** Variance explained by the individual coordinates, color coded by index measure. Others: refugees from Albania, Armenia, Chechnya, India, Kosovo, Lebanon, Somalia, Turkey, and Yemen

An unsupervised principal coordinate analysis on the Bray-Curtis and Jaccard distance matrices resulted in largest differences when comparing refugees to German control individuals, while regional differences between the microbiota of refugees are less prominent (Fig. 5). The permutational multivariate analysis of variance (PERMANOVA) test further validated the observation that German and refugee populations differ significantly in microbial composition and structure (Jaccard distances: *p* = 0.00021, Bray-Curtis: *p* = 0.00021; for details, please refer to Additional file 1: Table S9).

### Culture-based results on MDROs in swab samples from refugees

Overall, 506 screening swab specimens were inoculated on selective screening media for microbiological investigations. Of the 506 refugees screened, 6.3% (*n* = 32) and 1.6% (*n* = 8) were found to be colonized with MRSA and ESBL, respectively, by standard culture methods. CRE or VRE was not observed. Hence, the overall MDRO prevalence was 7.9%. Among the ESBL-producing Enterobacteriaceae, *Escherichia coli* (*n* = 3), *Klebsiella pneumoniae* (*n* = 3), and *Proteus mirabilis* (*n* = 2) were identified. Furthermore, one isolate of *Escherichia coli* was found to be additionally resistant to quinolones (see Additional file 1: Table S10 for prevalences and supporting material 01 for MDRO findings in each individual).

## Discussion

### Interconnection of emerging antibiotic resistances and migration events

In September 2016, the United Nations released a declaration on antibiotic resistances, stating that infections with antibiotic-resistant pathogens are one of the biggest known threats to humanity today [2]. This declaration is based on the recent Wellcome Trust report on antibiotic resistances [19], which is in agreement with the WHO and supports predictions of a post-antibiotic era in which even surgical standard procedures like cesarean section may become too dangerous to perform [5]. The development of antibiotic resistances is highly dependent on patterns of antibiotics use: countries where antibiotics are freely available (e.g., Arabian countries, large parts of the developing world) show larger problems than those with prescription restrictions [14].

Massive migration events have the potential to reshape the global distribution of antibiotic resistance genes. As a result of the Syrian crisis, more than 4.2 million refugees from Syria left the country [8]. More than 3.7 million refugees mainly from Syria, Afghanistan, and Iraq applied for asylum in Germany in the last 5 years [7]. With a steep north-south gradient for antibiotic resistance prevalences in Europe [6], migration of refugees may become an important factor for emerging antibiotic resistances in the Western world and may require employing rapid diagnostic methods upon contact with the healthcare system. While antibiotic resistance genes imported by single tourists are usually lost upon contact with the large remaining normal population, it is unclear how the massive import of complex resistomes changes the host country’s population microbiota.

### Increased prevalence of antibiotic resistance genes in refugees

Comparing antibiotic resistance gene prevalences in refugees from Syria, Afghanistan, Iraq, and neighboring countries to German control individuals, we observed three major categories of differences: (i) antibiotic resistance genes which show a high prevalence (> 90%) in all countries, including Germany, such as ermB, which was previously reported to be very frequent, independent of location [10, 20]; (ii) vanB, an antibiotic resistance gene exhibiting a higher prevalence in Germany when compared to refugees, which can be explained by its broad application in German agriculture and healthcare [21]; and (iii) a large number of antibiotic resistances which either show a drastically increased prevalence when compared to Germany or are not present at all in German control individuals. The first two categories of differences support the validity of or approach by confirming previous observations, while the third category—the increased prevalence in refugees—was expected due to considerable differences in consumption of and access to antibiotics: in Germany, the estimated percentage of antibiotics obtained without prescription was reported to be 0–3% [22]. These numbers increase drastically when moving southeast, starting in Greece with 10–15% [22], to Turkey with 44% [23], while data from Iran estimates rates of antibiotics obtained without prescription to be up to 58% [13]. Although no data on antibiotics obtained without prescription is available for most other countries as part of this study, one can assume rates to be in a range comparable to Iran, providing a plausible explanation for the increased prevalences we observed. At the same time, this is in line with our observation that the majority of Germans carry three or less antibiotic resistance genes, while Syrians carry mostly five or more antibiotic resistance genes per individual.

It is unclear which consequences the migration of individuals with higher antibiotic resistance gene prevalence may have on the local population, yet a few studies indicate potential risks: US military and civilian personnel serving in Iraq and Afghanistan between 2003 and 2005 suffered from infections with Acinetobacter strains, harboring OXA-23 and OXA-58 carbapenemases, which are uncommon in the USA [24]. Similarly, traveling to countries with higher antibiotic resistance prevalence may represent a risk to acquire resistances present in these countries, as shown for UK citizens that recently traveled India and Pakistan and can also introduce new antibiotic resistances which were previously not present in the country [25]. In contrast to that, resistance genes with similar prevalences in both countries, for example ermB, are not affected: they remain stable before and after travel [26], which matches our observation on the distribution of ermB. Interestingly, we observed that copy numbers of antibiotic resistance genes remained relatively stable across individuals of different origins, yet the corresponding prevalences did not exhibit such a stability. This could indicate an underlying, gene-specific mechanism, which has not been examined before.

In this context, it is important to take the heterogeneous nature of the cohort into account: different social and ethnic influences, different migration histories, and different exposures to adverse conditions are examples of factors potentially influencing the microbiome and the resistome. While these factors cannot be controlled in such a study design, the primary observations of prevalence differences are independent of individual outliers.

### Microbiota differences reflect different prevalences of antibiotic resistance genes

The uncontrolled access to antibiotics in developing countries is known to have an impact on the resistome of each individual, even without consuming antibiotics personally [27]. Together with the different lifestyles [28, 29], this may be the strongest factors shaping the microbiota including the resistome [14]. This is in line with our observation that the largest differences are found when comparing the microbiota between German control individuals and refugees, which was paralleled by our findings on the resistome. In this context, we found Firmicutes and Actinobacteria to be significantly more abundant in the German population, both of which are known to play an important role in the normal gut physiology [30]. In contrast to that, Bacteroidetes, a known pathobiont [31], was found to be increased in refugees. At the same time, Bacteroidetes represents a strong link to the antibiotic consumption since it has been previously reported to increase in response to antibiotic treatment [32]. The most prominent finding, however, is the significantly elevated load of Proteobacteria in refugees from Syria, Iraq, and Afghanistan. This group consists of many known human pathogens, for example *Klebsiella pneumoniae*, *Haemophilus influenzae*, and *Shigella sonnei*, which we found in refugees exclusively. It is important to note that out of the 67 refugee-associated indicator phylotypes identified, only seven could be categorized as signatures of potential pathogens. Anthropogenic use of antibiotics enriches the antibiotic resistance gene repertoire; however, the healthy human gut commensal microbiota is intrinsically loaded with antibiotic resistance genes. Functional metagenomic studies identified diverse antibiotic resistance genes in healthy adults [10] and children [33]. While we want to point out that most studies reflect the use of antibiotics in the US healthcare, data from Germany creates a different picture: here, only 12% of children up to the age of 4 years received two or more antibiotic treatments [34]—in contrast to the USA, where children of similar age receive, on average, at least one treatment pear year [35]. Likewise, antibiotic-naïve healthy infants within the first 2 months of age harbor antibiotic resistance genes [36]. In fact, we detected three antibiotic resistances genes in the stool of the only study participant younger than 1 year. While age has a substantial impact on the microbiota and the resistance profile [37], our study setup does not allow to draw conclusions about age-resistome interactions, since the cohort presented here consists of individuals with a median age of 25 years with only three children below the age of 10 (0, 6, and 9 years). Considering that the microbiome and the resistome are closely interconnected and keeping in mind that antibiotic resistances are present in antibiotic-naïve populations [38], it remains speculative whether the ARGs observed were originally acquired from pathogens or were intrinsic to commensals.

### Antibiotic resistance genes occur independent of signatures of potential pathogens

Antibiotic resistance genes did not correlate to potential pathogens identified. In contrast to that, we observed that bacterial taxa, which are associated to a decreased ARG load in German individuals, do not exhibit this effect in refugees. In this context, it is important to take the different nature of the results of culture-based and culture-independent methods into consideration. This lack of correlation is consistent with the observation that antibiotic resistance genes often occur independent of potentially pathogenic species but may also be found in the native commensal microbiota, which can serve as a reservoir for ARGs. Naturally, to confirm pathogenicity, further characterization would be required. The high prevalences of antibiotic resistance genes observed in refugees might represent a relevant reservoir for potential horizontal gene transfer, especially since the human intestinal microbiota, which is partially represented in the stool samples employed here, is known to be one of the most complex microbial communities in humans, where horizontal gene transfer can occur frequently [39]. Metagenomic approaches [40, 41] are often used to profile gut resistomes in infants [42] and adults [43, 44]. Culture-based approaches coupled with genome sequencing have identified ARGs in *Bifidobacteria* [45] and *Lactobacillus* [46] groups, both of which are common residents of the adult and infant gut and widely used as probiotics. In silico analysis on Bifidobacterial genomes predicted a substantial number of antibiotic resistance genes in the vicinity of mobile elements, enabling horizontal gene transfer in commensal gut microbiota. In the current study, we have implemented a culture-independent method to detect relevant antibiotic resistance genes irrespective of their host. As we did not monitor genes or mobile elements in the vicinity of ARGs, our data does not allow to draw conclusions about events like horizontal gene transfer, yet we believe that further assessment of this effect is urgently needed.

## Conclusions

Taken together, the data presented for the first time enables a quantitative comparison of antibiotic resistance genes and microbiota in refugees from Syria, Iraq, Afghanistan, and neighboring countries to individuals from Central Europe. The salient findings are the high prevalence of antibiotic resistance genes in refugees, large differences in the microbiota, and the observation that antibiotic resistance genes occur independent of potential pathogens. The potential consequences for healthcare systems of host countries warrant a careful and systematic evaluation of the natural course and impact of resistomes in the clinical and outpatient setting.

## Additional file

Additional file 1: Supplemental Material and Methods. (DOCX 1405 kb)

## Abbreviations

16S: 16S ribosomal RNA gene
ACC: Ambler class C beta-lactamases
ACT/MIR: act/mir gene (bacterial)
ampC: Ampicillin C-type β-lactamases
ARG: Antibiotic resistance gene
BIL/LAT/CMY: BIL/LAT/CMY-type β-lactamases
blaCTX: bla(CTX-M) extended-spectrum β-lactamase gene
blaOXA: bla(OXA-M) extended-spectrum β-lactamase gene
CRE: Carbapenems
CTX-M: CTX-M β-lactamase gene
DHA: DHA-type β-lactamase gene
ErmA: Erythromycin A resistance gene
ErmB: Erythromycin B resistance gene
ESBL: Gram-negative bacteria resistant to extended-spectrum β-lactams
EUCAST: European Committee on Antimicrobial Susceptibility Testing
femA SA: Aminoacyltransferase FemA gene
FOX: FOX-type ampicillin resistance gene
GAPDH: Glyceraldehyde 3-phosphate dehydrogenase
GES: GES-type extended-spectrum beta-lactamase gene
IMP: IMP-Type metallo-beta-lactamase gene
KPC: *Klebsiella pneumoniae* carbapenemase gene
MDRO: Multidrug-resistant organism
mefE: Macrolide resistance gene
MOX: MOX beta-lactamase gene
MRSA: Methicillin-resistant *Staphylococcus aureus*
NDM: New Delhi metallo-beta-lactamase gene
OXA: OXA β-lactamase gene
P2N: PopGen 2.0 Network
PER: PER β-lactamase gene
qnrA: Quinolone resistance gene A
qnrB: Quinolone resistance gene B
SHV: SHV β-lactamase gene
TEM: TEM β-lactamase gene
UNHCR: United Nations High Commissioner for Refugees
vanA: VanA-type vancomycin resistance gene
vanB: VanB-type vancomycin resistance gene
vanC: VanC-type vancomycin resistance gene
VEB: VEB β-lactamase gene
VIM: Verona integron-encoded metallo-beta-lactamase
VRE: Vancomycin-resistant enterococci
WHO: World Health Organization
