# Supplementary material for: The antibiotic resistome and microbiota landscape of refugees from Syria, Iraq and Afghanistan in Germany
Source: Microbiome. 2018 Feb 20;6:37. doi: 10.1186/s40168-018-0414-7 (PMC5819293; doi:10.1186/s40168-018-0414-7)
Supplement: Supplementary file 1 — Supplemental Material and Methods. (DOCX 1405 kb) [file 40168_2018_414_MOESM1_ESM.docx]

# Supplemental Material for “The antibiotic resistome and microbiota landscape of refugees from Syria, Iraq and Afghanistan in Germany” by Häsler et al.

# Supplemental Material and Methods

## Recruitment of participants and sample collection: further details

.

In the course of first health examination physicians or guided medical students took two swab specimen from three screening sites: one combined swab (FLOQSwab™, Copan, Brescia, Italy) of both anterior nares and the throat and a second skin swab from the groin area. Both swab tips were placed together in one sterile tube (Greiner bio-one, Frickenhausen, Germany) containing 2 ml of sterile sodium chloride 0.85 % solution. The swab specimen were stored at 4°C and further processed within up to 14 h in the microbiology laboratory of the University Medical Center Schleswig-Holstein, Kiel. In a first step, swabs were vortexed vigorously for 20 s in the sample tubes to suspend the patient material. Swab tips were discarded and sample suspensions were divided as follows: a) 100µl aliquots were used without any further dilution to inoculate selective agar plates for standard bacterial pathogen cultivation and b) 1ml was used for DNA extraction and subsequent qPCR-based TaqMan^®^ Array Micro Fluidic Card assay.

## DNA extraction from stool and swab samples: further details

Each swab-derived sample suspension was subjected to a rapid heat lysis protocol. Briefly, 1 ml of each sample suspension was centrifuged for 9 min at 3000 rpm followed by 1 min at 5000 rpm to settle down viable bacteria. The supernatant was discarded and 120 µl of lysis buffer (10 mM Tris-HCl pH 8, 1 mM EDTA, 1 % Triton-X 100, 0.5 % Tween 20) were added. The tube was vortexed vigorously for 15 s, followed by a heating step on a Thermomixer® (Eppendorf, Germany) for 11 min at 99 °C and 900 rpm. Lysed suspensions were frozen at -80 °C until qPCR was performed in batches.

Stool samples were thawed and approximately 0.2 g were used for DNA extraction with the MoBio PowerSoil® DNA Isolation Kit (Mo Bio Laboratories, Inc., Carlsbad, USA) according to the protocol suggested by the manufacturer. Extracted DNA was eluted from the spin filter silica membrane with 100 µl of elution buffer and stored at -80 °C.

## Microbiota profiling using 16S rRNA gene sequencing, further details:

In order to profile bacterial communities in stool and swab samples, DNA was extracted as described above. 16S rRNA gene variable region V3-4 was amplified using the dual barcoded bacterial specific primer pair 341F (GTGCCAGCMGCCGCGGTAA) and 806R (GGACTACHVGGGTWTCTAAT). Each primer contains a 12 base Golay barcode, Illumina adaptor, primer pad, linker and 16S rRNA gene specific sequences. An aliquot (2 µl) of DNA was amplified using composite primers (as above) in duplicate PCR reactions in a GeneAmp PCR system 9700 (Applied Biosystems, Foster City, California, USA). PCR was performed with the following cycling conditions; an initial denaturation of 3 min at 98°C, followed by 30 cycles, 98°C for 10 seconds, at 55°C for 30 seconds, 72°C for 30 seconds, and a final extension at 72°C for 10 minutes. Amplified products from each samples were visualized on agarose gel to control the performance of the PCR reactions. Amplicon quantities were normalized by employing SequalPrep kit (Applied Biosystems/Thermo Fisher Scientific, Darmstadt, Germany). Sterile PCR grade water was used as a negative control. But we failed to detect any amplification in PCR reaction from these templates. Equal volumes (SequalPrep normalzed) of amplicons were pooled to prepare a library. Sequencing was performed using an Illumina MiSeq (2 x 300 sequencing kit).

## Obtained 300 base paired reads were processed for quality control using the software mother [1]. Forward and reverse reads were assembled to form contigs. Contigs larger than 450 bases in length, having any ambiguous base (N), and/or more than 8 homopolymers were identified and discarded from downstream analysis. Subsequently the remaining reads were subjected to alignment against mothur curated SILVA (release 128) reference alignment. Reads not aligning to this reference alignment in V3-V4 region were also removed from analysis. Potentially chimeric reads were detected with Uchime algorithm [2] and removed. Sequences were classified using mothur-curated greengenes training sets (fasta and taxonomy). Sequences classified as eukaryotes, chloroplasts and mitochondria were removed from the analysis. Remaining good quality sequences were binned in phylotypes against the greengenes reference.”

References for microbiota profiling via 16S rRNA gene sequencing:

1. Schloss, Patrick D., Sarah L. Westcott, Thomas Ryabin, Justine R. Hall, Martin Hartmann, Emily B. Hollister, Ryan A. Lesniewski, et al. “Introducing Mothur: Open-Source, Platform-Independent, Community-Supported Software for Describing and Comparing Microbial Communities.” *Appl. Environ. Microbiol.* 75, no. 23 (December 1, 2009): 7537–41. <https://doi.org/10.1128/AEM.01541-09>.
2. Edgar, Robert C., Brian J. Haas, Jose C. Clemente, Christopher Quince, and Rob Knight. “UCHIME Improves Sensitivity and Speed of Chimera Detection.” *Bioinformatics (Oxford, England)* 27, no. 16 (August 15, 2011): 2194–2200. https://doi.org/10.1093/bioinformatics/btr381.

## Detection of antibiotic resistance genes via microfluidic real time PCR: further details

Here we employed a fast TaqMan® real-time PCR approach targeting 37 of the most common resistance determinants in MRSA, VRE, ESBL and CRE, supplemented by three additional species-specific gene targets and five assays targeting resistance genes conferring macrolide resistance in streptococci and quinolone resistance in Enterobacteriaceae. To ascertain assay quality one exogenous and two endogenous internal control assays were integrated on the TaqMan^®^ Array Micro Fluidic Cards (TAC). Adequate sample lysis was monitored by targeting the human GAPDH gene and the bacterial 16S rRNA gene as human and bacterial housekeeping genes, respectively. Inhibition of amplification was monitored by targeting an artificial exogenous internal control (IPC). All target gene assays employed are listed in supplemental table 2.

Primers and probes of all TaqMan^®^ real-time PCR assays were preloaded on TACs by the manufacturer. TACs assigned to the same production lot were initially tested with nuclease free water as no template control and a positive control mix containing pooled target DNA from positive reference strains (for a list of reference strains, please see supplemental table 3).

## Detection of antibiotic resistance genes via microfluidic real time PCR: performance testing I

Bacterial isolates from fresh overnight cultures grown on Columbia blood agar were used for DNA extraction with a QIAamp® DNA Mini Kit according to the protocol suggested by the manufacturer. Extracted bacterial DNA was eluted from spin columns in 100 µl of elution buffer and stored at -20 °C. The nucleotide concentrations were determined using NanoDrop™ 1000 Spectrophotometer (Thermo scientific) and corresponding genome equivalents were calculated as described before (Rachwal PA, Rose HL, Cox V, Lukaszewski RA, Murch AL, Weller SA. The Potential of TaqMan Array Cards for Detection of Multiple Biological Agents by Real-Time PCR. PLoS ONE 2012; 7). The molecular weights of bacterial genomes where estimated based on published data from NCBI Genome (http://www.ncbi.nlm.nih.gov/genome).

Specificity of each integrated primer and probe set was preliminary assessed in individual qPCR reactions utilizing at least one strain containing the target gene (positive control) and a variety of related strains producing other or no resistance genes (negative control). Individual qPCR assays were run on the same ViiA™ 7 real-time PCR system and the presence/absence of the expected genes was determined.

Analytical sensitivity of the TAC assay employed here was estimated using dilution series of DNA extracts from a set of positive control strains for each target represented on the TAC. In order to eliminate interferences between strains harbouring the same resistance gene, two positive control mixtures (PCM) were prepared. Briefly, DNA extracts of non-interfering positive control strains were pooled to a final concentration of 4,000 GE per µl for each strain. A four-fold serial dilution of both mixtures was performed and port reservoirs were loaded with decreasing GE amounts from 200,000 GE in port one to 50 GE in port 7 obtaining a range of 2,000 GE to 0,5 GE per reaction volume (1 µl well). Port 8 was loaded with nuclease free water as NTC. Both PCMs were run separately on a single TAC.

## Detection of antibiotic resistance genes via microfluidic real time PCR: performance testing II

The TaqMan^®^ real-time PCR system was evaluated with surveillance specimens from 73 individuals who were previously found to be positive for MRSA, VRE, ESBL and CRE or active surveillance specimens from individuals on hospital admission with risk factors for MDRO colonization. Specimens comprise one combined swab of both anterior nares and the throat and a second swab from the perianal area. Sample processing compares to the refugee approach. For TaqMan^®^ real-time PCR, bacterial lysis deviates from the procedure described above. Briefly, 469 µl sample suspension were mixed with 31 µl of 16.1 times concentrated lysis buffer (161 mM Tris-HCl pH 8, 16.1 mM EDTA, 16.1 % Triton-X 100, 8.1 % Tween 20) to a final volume of 500 µl and vortexed vigorously for 15 s. Bacteria were heat lysed at 99 °C for 15 min. Lysed suspensions were frozen at -80 °C until qPCR was performed in batches. Results of real-time PCR were compared to those of routine culture and sensitivity, specificity, positive predictive value (PPV) and negative predictive value (NPV) were estimated.

## Analysis of the interaction between the microbiome and the resistome

As a measure of the interaction between individual microbial taxa and individual antibiotic resistance genes, a spearman rank correlation coefficient was employed. These correlation coefficients were further utilized as follows: i) Correlation pairs of microbial taxa and antibiotic resistances which showed a negative correlation in German individuals (taxa, categorized as ABR repelling) were assessed for their impact in refugees by counting the number of its positive and negative correlations. ii) The distribution of these correlation coefficients was assessed. This analysis was conducted for Germany, Syria, Iraq and Afghanistan. Other countries of origin had to be omitted due to the lower number of individuals (n≤20).

## Categorization of bacterial species into pathogens and non pathogens

Bacterial species were classified into pathogens and non pathogens based on the references listed in Supplemental table 5. *Escherichia coli* was not categorized as a pathogen since our data does not allow strain discrimination.

# Supplemental Figures

## Supplemental figure 1: Quantitative dependencies between prevalence and resistance gene copy numbers.

Selected antibiotic resistance genes are shown with their prevalence and the corresponding median gene copy number observed in carriers. Others: refugees from Albania, Armenia, Chechnya, India, Kosovo, Libanon, Somalia, Turkey and Yemen.

## Supplemental Figure 2: Alpha diversity indices assessing regional differences found in stool samples

*Box plots representing the distribution of alpha diversity indices in German controls and refugees from different countries. Whiskers represent the 5–95% percentiles.*

## Supplemental Figure 3: Interaction of microbial taxa and antibiotic resistance genes.

*A: Bacterial taxa which show a negative correlation (spearman rho) to antibiotic resistance genes in German individuals (thus categorized as antibiotic resistance repelling in Germany) do not show this effect in refugees (more positive correlations). Counts of correlations are plotted on the y-axis (negative correlations plotted below the x-axis), countries are colour coded. B: Distribution of correlation values for all bacterial taxa and specific antibiotic resistance genes. The counts are plotted on the y-axis, while the x-axis depicts the spearman-rho values. Countries are colour coded, for visualization purposes the counts of different regions were scaled. TEM, mefE and vanC1 show more negative correlations when comparing German individuals to refugees, while ermB is an example of a resistance gene not showing this effect.*

# Supplemental Tables

## Supplemental table 1: List of media employed for cultivation of multi drug resistant organisms (MDRO)

| **Media** | **MDRO** |
| --- | --- |
| ChromID MRSA (bioMérieux, Marcy-l’Étoile, France) | methicillin-resistant Staphylococcus aureus (MRSA), |
| chromID VRE (bioMérieux) | vancomycin-resistant Entero¬coccus ssp. (VRE) |
| chromID ESBL (bioMérieux) with a disk of imipenem 10 µg (Oxoid, Thermo Fisher Scientific, Waltham, USA). | multidrug-resistant Gram-negative bacteria expressing extended-spectrum β-lactamases (ESBL) and/or carbapenemases (CRE) |

## Supplemental table 2: List of all assays employed as part of the TaqMan® based microfluidic real time PCR system

| **Target** | **Resistance** | **Assay ID** | **Detection limit [GE]** | **Context Sequence** |
| --- | --- | --- | --- | --- |
| 16S | endogenous control | Pa04230899_s1 | N/A | CCGCAACGAGCGCAACCCTTATTGT |
| ACC | AmpC resistance | Pa04646144_s1 | 1 | CTGTTATCCGTGATTACCTGTCTGG |
| ACICU_00593 | Species specific, A. baumannii | Pa04646148_s1 | 2 | N/A |
| ACT/MIR | AmpC resistance | Pa04646124_s1 | 2 | ACCGTTACGCCGCTGATGAAAGCGC |
| ampC | AmpC resistance | Pa04646117_s1 | 2 | GCCGAAAGCGGAAGAGGCGCATTAC |
| BIL/LAT/CMY | AmpC resistance | Pa04646135_s1 | 1 | TAAGACGTTTAACGGCGTGTTGGGC |
| CTX-M group 1 | Extended spectrum beta-lactamase | Pa04646149_s1 | 2 | AATGGGACGATGTCACTGGCTGAGC |
| CTX-M group 2 | Extended spectrum beta-lactamase | Pa04646142_s1 | 8 | GACGCTACCCCTGCTATTTAGCAGC |
| CTX-M group 8/25 | Extended spectrum beta-lactamase | Pa04646154_s1 | 1 | CCCGCGCGATACCACCACGCCATTA |
| CTX-M group 9 | Extended spectrum beta-lactamase | Pa04646127_s1 | 32 | GCTTAATCAGCCTGTCGAGATCAAG |
| DHA | AmpC resistance | Pa04646120_s1 | 8 | CGCTGATAATGTCGCGGCGGTGGTG |
| ErmA | Macrolide resistance | Pa04646137_s1 | N/D | ATTCTAAAATTTTCCTTCCCAAAAC |
| ErmB | Macrolide resistance | Pa04230913_s1 | 1 | AAACTTACCCGCCATACCACAGATG |
| exoIPC | inhibition control, exogenous | Ac04646146_a1 | N/A | TCTCGTTGATCTTCCGTTGATAGTG |
| femA SA | Species specific, S. aureus | Pa04646123_s1 | 2 | GCAGTGCAATGGGAAATGATTAATT |
| femA SE | Species specific, S. epidermidis | Pa04646141_s1 | 32 | TTCAAATCGTTATCGCCATTTTGCA |
| FOX | AmpC resistance | Pa04646126_s1 | N/D | CTGGGCCAGCCATTTGAGCAACTGA |
| GAPDH | endogenous control | Hs99999905_m1 | N/A | N/A |
| GES | Extended spectrum beta-lactamase | Pa04646151_s1 | N/D | ACGTGACGAATTAGTTGCCTCTGTC |
| IMP-1 group | Carbapenem resistance | Pa04646131_s1 | 1 | ACTCCATTTACGGCTAAAGATACTG |
| IMP-16 | Carbapenem resistance | Pa04646116_s1 | 1 | AGACGGTAAGGTGCAAGCTAAAAAC |
| IMP-2 group | Carbapenem resistance | Pa04646119_s1 | 2 | TCACATTTCCATAGCGACAGCACGG |
| IMP-7 | Carbapenem resistance | Pa04646158_s1 | 1 | TTAATATCCAAATATGGTAAGGCAA |
| KPC | Carbapenem resistance | Pa04646152_s1 | 2 | CCTCGTCGCGGAACCATTCGCTAAA |
| mefE | Macrolide resistance | Pa04230904_s1 | 2 | AAAACAGGATCTGCGATGGTCTTGT |
| MOX/CMY | AmpC resistance | Pa04646156_s1 | N/D | CATCCAGCCGCTGCTCAAGGAGCAC |
| NDM | Carbapenem resistance | Pa04646121_s1 | 2 | GATTGCCGAGCGACTTGGCCTTGCT |
| OXA-1 | Extended spectrum beta-lactamase | Pa04646133_s1 | 2 | TCATACACCAAAGACGTGGATGCAA |
| OXA-23 | Carbapenem resistance | Pa04646139_s1 | 1 | TCAAGAGGTAGAGTTTGTTTCCCAA |
| OXA-40 | Carbapenem resistance | Pa04646143_s1 | 8 | GGATGGGTTGTTCAGCCTCAAGGGA |
| OXA-48 | Carbapenem resistance | Pa04646138_s1 | 2 | TACCCGCATCTACCTTTAAAATTCC |
| OXA-58 | Carbapenem resistance | Pa04646136_s1 | 2 | TGGGATGGAAAGCCACGTTTTTTTA |
| OXA-72 | Carbapenem resistance | Pa04646118_s1 | 1 | CCGATGACCTTGCACATAACCGATT |
| PER-1 | Extended spectrum beta-lactamase | Pa04646140_s1 | N/D | AACAGTGGGGATTGCGCTGAGGTTT |
| PER-2 | Extended spectrum beta-lactamase | Pa04646157_s1 | N/D | GGTTGAAACCACCACAGGACCACAG |
| QnrA | Quinolone and fluoroquinolone resistance | Pa04646160_s1 | N/D | AAGCGGCGCCGTTGAAGGGTGCCAC |
| QnrB | Quinolone and fluoroquinolone resistance | Pa04646145_s1 | N/D | N/A |
| SHV | Extended spectrum beta-lactamase | Pa04646134_s1 | 1 | CGCTTTCCCATGATGAGCACCTTTA |
| TEM | Extended spectrum beta-lactamase | Pa04646128_s1 | 32 | GCGGCCAACTTACTTCTGACAACGA |
| vanA1 | Vancomycin resistance | Pa04646159_s1 | 8 | GACTACGCAATTGAATCGGCAAGAC |
| vanA2 | Vancomycin resistance | Pa04646147_s1 | 1 | AGCTACTCCCGCCTTTTGGGTTATT |
| vanB | Vancomycin resistance | Pa04646150_s1 | 1 | AACTTAACGCTGCGATAGAAGCGGC |
| vanC1 | Vancomycin resistance | Pa04646130_s1 | 2 | CAATTGACGATTGGTGCTTGTGATG |
| vanC2-C3-1 | Vancomycin resistance | Pa04646129_s1 | N/D | TCACAAAAGTAACTTGTGTTGAAGA |
| vanC2-C3-2 | Vancomycin resistance | Pa04646122_s1 | 1 | TTGAGATCGGTTGCGGTATTTTGGG |
| VEB | Extended spectrum beta-lactamase | Pa04646153_s1 | N/D | ACCGATTAAAAGGACAATTACCAAA |
| VIM | Carbapenem resistance | Pa04646155_s1 | 2 | GATGGTGATGAGTTGCTTTTGATTG |

Detection limit was calculated as genome equivalents per TAC reaction volume of the bacterial reference strain carrying the resistance gene; N/A not applicable; N/D not detected;

## Supplemental table 3: List of reference strains used for performance assessment of the microfluidic system employed

| species | strain ID | genotype | source |
| --- | --- | --- | --- |
| Acinetobacter baumannii | NCTC 13301 | OXA-23, OXA-51-like | NCTC |
| Acinetobacter baumannii | NRZ-00330 | OXA-23 | InfMed |
| Acinetobacter baumannii | NRZ-00518 | OXA-58 | InfMed |
| Acinetobacter baumannii | NRZ-00449 | OXA-72 | InfMed |
| Enterobacter asburiae | NRZ-01112 | MIR | RUB |
| Enterobacter cloacae | NCTC 13405 | AmpC | NCTC |
| Enterobacter cloacae | NCTC 13406 | AmpC | NCTC |
| Enterobacter cloacae | NCTC 13464 | CTX-M group 9 | NCTC |
| Enterobacter cloacae | NRZ-00239 | VIM-1 | RUB |
| Enterobacter cloacae | NRZ-00239 | VIM-1 | InfMed |
| Enterococcus casseliflavus | RKI UW703 | VanC-2 | InfMed |
| Enterococcus faecalis | ATCC 29212 |  | InfMed |
| Enterococcus faecalis | ATCC 51299 |  | InfMed |
| Enterococcus faecalis | NCTC 12201 | VanA | NCTC |
| Enterococcus faecium | RKI UW2314 | VanA | RKI |
| Enterococcus faecium | RKI UW699 | VanB | RKI |
| Enterococcus faecium^2)^ | clinical isolate | | InfMed |
| Enterococcus gallinarium | RKI UW701 | VanC-1 | RKI |
| Escherichia coli^3)^ | ATCC 11775 |  | InfMed |
| Escherichia coli | 14/05 | CTX-M 14, | RKI |
| Escherichia coli | 129/07 | CTX-M 2, TEM-1 | RKI |
| Escherichia coli | NRZ-8814 | CTX-M group 8 | RUB |
| Escherichia coli | NRZ-02721 | TEM-1, OXA-1, OXA-10 | RUB |
| Escherichia coli | NCTC 13461 | CTX-M group 1 | NCTC |
| Escherichia coli | NCTC 13462 | CTX-M group 2 | NCTC |
| Escherichia coli | NCTC 13463 | CTX-M group 8 | NCTC |
| Escherichia coli | NRZ-00401 | VIM-1 | InfMed |
| Escherichia coli | NRZ-00302 | NDM-1 | InfMed |
| Klebsiella oxytoca | 106/04 | CTX-M 9 | RKI |
| Klebsiella oxytoca | NRZ-01745 | VIM-1, ACC | RUB |
| Klebsiella oxytoca | NRZ-00405 | VIM-1 | InfMed |
| Klebsiella pneumoniae | UR 1697 | VIM | InfMed |
| Klebsiella pneumoniae | ESBL 25 | CTX-M 3, TEM-1, SHV-12 | InfMed |
| Klebsiella pneumoniae | ESBL 299 | CTX-M 2, TEM-1, SHV-5 | InfMed |
| Klebsiella pneumoniae | ESBL 302 | CTX-M 14, [-] TEM, SHV-11 | InfMed |
| Klebsiella pneumoniae | ESBL 344 | CTX-M 3, [-] TEM, [+] SHV | InfMed |
| Klebsiella pneumoniae | ESBL 356 | CTX-M 15, TEM-1, SHV-1 | InfMed |
| Klebsiella pneumoniae | ESBL 398 | CTX-M 15, TEM-1, SHV-1 | InfMed |
| Klebsiella pneumoniae | ESBL 538 | [-] CTX-M, TEM-3, SHV-38 | InfMed |
| Klebsiella pneumoniae | 24/10 | KPC | RKI |
| Klebsiella pneumoniae | NRZ-00002 | OXA-48 | RUB |
| Klebsiella pneumoniae | NRZ-00103 | KPC-2 | RUB |
| Klebsiella pneumoniae | NRZ-00313 | NDM-1 | RUB |
| Klebsiella pneumoniae | NRZ-01013 | CMY-2 | RUB |
| Klebsiella pneumoniae | NRZ-06269 | DHA-1 | RUB |
| Klebsiella pneumoniae | NCTC 13465 | CTX-M group 25 | NCTC |
| Klebsiella pneumoniae | NRZ-00535 | VIM-1 | InfMed |
| Klebsiella pneumoniae | NRZ-00103 | KPC-2 | InfMed |
| Klebsiella pneumoniae | NRZ-00223 | KPC-3 | InfMed |
| Klebsiella pneumoniae | NRZ-00002 | OXA-48 | InfMed |
| Klebsiella pneumoniae | NRZ-05050 | OXA-48, OXA-1 | InfMed |
| Klebsiella pneumoniae | NRZ-05747 | OXA-48 | InfMed |
| Klebsiella pneumoniae | NRZ-05782 | OXA-48, TEM | InfMed |
| Proteus mirabilis | NRZ-00185 | CMY-2 | InfMed |
| Pseudomonas aeruginosa | ATCC 10145 |  | InfMed |
| Pseudomonas aeruginosa | NRZ-00196 | IMP-16 | RUB |
| Pseudomonas aeruginosa | NRZ-00425 | VIM-2 | RUB |
| Pseudomonas aeruginosa | NRZ-00638 | IMP-7 | RUB |
| Pseudomonas aeruginosa | NRZ-01058 |  | RUB |
| Pseudomonas aeruginosa | CF446 |  | RUB |
| Pseudomonas aeruginosa | 48/09 | IMP-2 | RKI |
| Pseudomonas aeruginosa | NRZ-00425 | VIM-2 | InfMed |
| Staphylococcus aureus | ATCC 12600 |  | InfMed |
| Staphylococcus aureus | ATCC 29213 |  | InfMed |
| Staphylococcus aureus | ATCC 43300 | mecA | InfMed |
| Staphylococcus epidermidis | ATCC 12228 |  | InfMed |
| Staphylococcus epidermidis | ATCC 14990 |  | InfMed |
| Staphylococcus epidermidis | 12058814-01 | mecA | InfMed |
| Staphylococcus haemolyticus | ATCC 29970 |  | InfMed |
| Staphylococcus hominis | ATCC 27844 |  | InfMed |
| Staphylococcus saprophyticus | ATCC 15305 |  | InfMed |
| Streptococcus mitis | NRZ-57642 | ermB | RWTH |
| Streptococcus mitis | NRZ-57099 | mefE | RWTH |
| Streptococcus mitis | NRZ-57641 | mefE | RWTH |
| Streptococcus mitis | NRZ-56156 |  | RWTH |
| Streptococcus mitis | NRZ-56752 |  | RWTH |
| Streptococcus mitis | NRZ-56767 |  | RWTH |
| Streptococcus pneumoniae | NRZ-23083 |  | RWTH |
| Streptococcus pneumoniae | NRZ-23090 |  | RWTH |
| Streptococcus pneumoniae | NRZ-23092 |  | RWTH |
| Streptococcus pneumoniae | NRZ-46335 | ermB | RWTH |
| Streptococcus pneumoniae | NRZ-50330 | ermB | RWTH |
| Streptococcus pneumoniae | NRZ-59543 | ermB | RWTH |
| Streptococcus pneumoniae | NRZ-43901 | mefA | RWTH |
| Streptococcus pneumoniae | NRZ-44908 | mefA | RWTH |
| Streptococcus pneumoniae | NRZ-56298 | mefA | RWTH |
| Streptococcus pneumoniae | NRZ-42588 | mefE | RWTH |
| Streptococcus pneumoniae | NRZ-58434 | mefE | RWTH |
| Streptococcus pneumoniae | NRZ-58538 | mefE | RWTH |
| Streptococcus pneumoniae | NRZ-43994 | mefE, ermB | RWTH |
| Streptococcus pneumoniae | NRZ-49732 | mefE, ermB | RWTH |
| Streptococcus pneumoniae | NRZ-52410 | mefE, ermB | RWTH |

InfMed = Institute of Infection Medicine, Christian-Albrecht University of Kiel, Brunswiker Straße 4,
24105 Kiel, Germany (S. Schubert and R. Podschun)

NCTC = National Collection of Type Cultures, England

RUB = National Reference Laboratory for multidrug-resistant gram-negative bacteria for Germany, Institute of Medical Microbiology at the Ruhr-University Bochum, Universitätsstr. 150,
44801 Bochum, Germany (S. Gatermann and M. Kaase)

RKI = The Robert Koch Institute, Burgstr. 37, 38855 Wernigerode, Germany, Department 1, Unit 12 Nosocomial Pathogens and Antibiotic Resistances (G. Werner and Y. Pfeifer)

RWTH = National Reference Laboratory on Streptococcal Diseases, Institute of Medical Microbiology at the RWTH University Hospital, Pauwelstraße 30, 52074 Aachen, Germany (M. van der Linden)

## Supplemental table 4: Clinical validation of TaqMan Real-Time PCR assays based on swab samples

|  | **number of patients tested** | **culture positive** | **real-time PCR positive** | **sensitivity**  **[%]** | **specificity**  **[%]** | **PPV**  **[%]** | **NPV**  **[%]** |
| --- | --- | --- | --- | --- | --- | --- | --- |
| MRSA | 73 | 9 | 3 | 33,3 | 100,0 | 100,0 | 91,4 |
| VRE | 72 | 13 | 15 | 84,6 | 93,2 | 73,3 | 96,5 |
| ESBL | 72 | 8 | 32 | 75,0 | 59,4 | 18,8 | 95,0 |
| CRE | 72 | 1 | 1 | 100,0 | 100,0 | 100,0 | 100,0 |

PPV: positive predictive value, NPV: negative predictive value

## Supplemental table 5: Categorization of potential human pathogens found in 16S rRNA gene sequencing data and corresponding references

| **Species** | **Transmission** | **Diseases** |
| --- | --- | --- |
| Chlamydia pneumoniae | Respiratory droplets[1][2], vaginal sex[1], oral sex[1], anal sex[1] Vertical from mother to newborn (ICN)[1], Direct or contaminated surfaces and flies (trachoma)[1] | Atypical pneumonia[2], Trachoma[1][2], Neonatal conjunctivitis[1][2], Neonatal pneumonia[1][2]. |
| Clostridium perfringens | Spores from soil,[1][2] persevere in canned food, smoked fish and honey[2], Gut flora,[1][2] overgrowing when other flora is depleted[1], Spores in soil[1][2], Vaginal flora and gut flora[1], Spores in soil, skin penetration through wounds[1][2] | Anaerobic cellulitis[1][2], Gas gangrene[1][2] Acute food poisoning[1][2] |
| Haemophilus influenzae | Droplet contact[1], Human flora of e.g. upper respiratory tract[1] | Bacterial meningitis[1][2], Upper respiratory tract infections[1][2], Pneumonia,[1][2] bronchitis[1], Septic arthritis in infants[2] |
| Helicobacter pylori | Colonizing stomach[1], Unclear person-to-person transmission[1] | Peptic ulcer[1][2], Chronic gastritis[2], Risk factor for gastric carcinoma and gastric B-cell lymphoma[1] |
| Klebsiella pneumoniae | Mouth, skin, and gut flora.[3], Pneumonia upon aspiration | Klebsiella pneumonia, with significant lung necrosis and hemoptysis[2], Hospital-acquired Urinary tract infection and sepsis[2] |
| Legionella pneumophila | Droplet contact, from e.g. cooling towers,[1][2] humidifiers,[1] air conditioners[1][2] and water distribution systems[1] | Legionnaire's Disease[1][2], Pontiac fever[1][2] |
| Shigella sonnei | Faecal-oral[1][2] | Shigellosis (bacillary dysentery) |
| Shigella dysenteriae | Faecal-oral[1][2] | Shigellosis (bacillary dysentery) |
| Staphylococcus aureus | Human flora on mucosae in e.g. anterior nares, skin and vagina,[1][2] entering through wound | Coagulase-positive staphylococcal infections (Skin infections, including impetigo[1][2], Acute infective endocarditis[1][2], Sepsis[1][1], Necrotizing pneumonia[1], Meningitis[2], Osteomyelitis[2]), Toxinoses (Scalded skin syndrome[1][2], Toxic shock syndrome[1][2], Staphylococcal food poisoning[1][2]) |
| Staphylococcus epidermidis | Human flora in skin,[1][2] anterior nares[1] and mucous membranes[2] | Infections of implanted prostheses (e.g. heart valves[1] and joints[2]) and catheters[1][2] |
| Staphylococcus saprophyticus | Part of normal vaginal flora[1] | Cystitis in women[1][2] |
| Streptococcus agalactiae | Human flora in vagina,[1][2] urethral mucous membranes,[1] rectum[1], Vertically during childbirth[1], Sexually[1] | Neonatal meningitis[1][2], Neonatal sepsis[1][2], Neonatal pneumonia[2], Endometritis in postpartum women[1], Opportunistic infections with septicemia and pneumonia[1] |

References for categorization of human pathogens

1. Fisher, Bruce; Harvey, Richard P.; Champe, Pamela C. (2007). Lippincott's Illustrated Reviews

2. "Bacteria Table" (PDF). Creighton University School of Medicine. Retrieved 2015-05-03.

3. Ryan, KJ; Ray, CG, eds. (2004). Sherris Medical Microbiology (4th edition). McGraw Hill. ISBN 0-8385-8529-9.

## Supplemental table 6: Ten most prevalent antibiotic resistance genes and pathogens in combined screening swab specimens from both nostrils, the throat and the groin

|  | **Prevalence [%]/total number of findings** | | | | | |
| --- | --- | --- | --- | --- | --- | --- |
|  | Syria | Iraq | Iran | Afghanistan | Eritrea | others |
|  | n=235 | n=115 | n=20 | n=95 | n=20 | n=20 |
| **Antibiotic resistance gene** | | | | | | |
| mefE | 100.0/235 | 100.0/115 | 100.0/20 | 100/95 | 95.0/19 | 95.0/19 |
| ermB | 99.6/234 | 100.0/115 | 90.0/18 | 97.9/93 | 80.0/16 | 85.0/17 |
| mecA | 90.2/21 | 93.9/108 | 95.0/19 | 85.3/81 | 90.0/18 | 70.0/14 |
| TEM | 29.4/69 | 27.8/3 | 20.0/4 | 16.8/16 | 20.0/4 | 25.0/5 |
| SHV | 6.8/16 | 7.0/8 | 0.0/0 | 6.3/6 | 0.0/0 | 10.0/0 |
| ermA | 3.0/7 | 2.6/3 | 0.0/0 | 6.3/6 | 0.0/0 | 5.0/1 |
| BIL/LAT/CMY | 2.6/6 | 0.9/1 | 0.0/0 | 4.2/4 | 0.0/0 | 5.0/1 |
| MOX/CMY | 2.6/6 | 0.9/1 | 0.0/0 | 0.0/0 | 0.0/0 | 0.0/0 |
| OXA-58 | 2.6/6 | 0.9/1 | 0.0/0 | 5.3/5 | 0.0/0 | 0.0/0 |
| CTX-M 1 group | 1.3/3 | 6.1/7 | 5.0/1 | 2.1/0 | 0.0/0 | 0.0/0 |
| **Pathogens** |  | | | | | |
| Haemophilus influenzae | 94.9/223 | 84.3/97 | 85.0/17 | 91.6/87 | 95.0/19 | 85.0/17 |
| Staphylococcus epidermidis | 80.4/189 | 74.8/86 | 60.0/1 | 74.7/71 | 80.0/16 | 70.0/14 |
| Escherichia coli | 14.5/34 | 13.0/15 | 10.0/0 | 21.1/20 | 20.0/4 | 15.0/3 |
| Staphylococcus aureus | 10.6/25 | 13.0/15 | 10.0/0 | 18.9/18 | 10.0/0 | 0.0/0 |
| Staphylococcus saprophyticus | 4.3/10 | 2.6/3 | 0.0/0 | 1.1/1 | 0.0/0 | 10.0/0 |
| Bacteroides fragilis | 3.4/8 | 2.6/3 | 5.0/1 | 5.3/5 | 10.0/0 | 5.0/1 |
| Streptococcus agalactiae | 3.0/7 | 3.5/4 | 20.0/4 | 3.2/3 | 0.0/0 | 5.0/1 |
| Legionella pneumophila | 2.6/6 | 0.9/1 | 0.0/0 | 0.0/0 | 0.0/0 | 0.0/0 |
| Clostridium perfringens | 0.0/0 | 0.9/1 | 0.0/0 | 0.0/0 | 0.0/0 | 0.0/0 |

## Supplemental table 7: Average relative distribution of bacterial phyla in stool samples from German individuals and refugees.

| **Phyla** | **Germany** | **Syria** | **Iraq** | **Iran** | **Afghanistan** | **Eritrea** | **Others** |
| --- | --- | --- | --- | --- | --- | --- | --- |
| Firmicutes | 71.42 | 45.33 | 43.94 | 44.10 | 42.80 | 44.01 | 47.30 |
| Actinobacteria | 14.84 | 6.96 | 6.77 | 10.76 | 7.62 | 6.05 | 5.90 |
| Bacteroidetes | 6.75 | 35.20 | 34.36 | 31.30 | 33.39 | 35.34 | 31.46 |
| Proteobacteria | 5.01 | 10.82 | 12.24 | 11.91 | 12.47 | 12.18 | 11.60 |
| Verrucomicrobia | 1.18 | 0.13 | 0.08 | 0.67 | 0.09 | 0.12 | 1.03 |
| Tenericutes | 0.32 | 0.17 | 0.31 | 0.12 | 0.32 | 0.11 | 0.75 |
| Bacteria_unclassified | 0.31 | 0.65 | 0.76 | 0.65 | 0.89 | 0.44 | 1.47 |
| Cyanobacteria | 0.14 | 0.29 | 0.36 | 0.14 | 0.72 | 0.03 | 0.29 |
| TM7 | 0.01 | 0.03 | 0.01 | 0.01 | 0.00 | 0.01 | 0.00 |
| Fusobacteria | 0.00 | 0.02 | 0.00 | 0.00 | 0.01 | 0.02 | 0.02 |
| Spirochaetes | 0.00 | 0.21 | 0.05 | 0.00 | 0.26 | 1.32 | 0.04 |
| Synergistetes | 0.00 | 0.00 | 0.03 | 0.02 | 0.00 | 0.01 | 0.04 |
| Lentisphaerae | 0.00 | 0.09 | 0.09 | 0.03 | 0.08 | 0.05 | 0.07 |
| Elusimicrobia | 0.00 | 0.11 | 1.01 | 0.29 | 1.34 | 0.03 | 0.02 |
| GN02 | 0.00 | 0.00 | 0.00 | 0.00 | 0.00 | 0.00 | 0.00 |
| WPS-2 | 0.00 | 0.00 | 0.00 | 0.00 | 0.00 | 0.26 | 0.00 |

## Supplemental table 8: P values obtained from pair-wise comparisons of major bacterial phyla from stool samples between German control and refugees.

Kruskall-Wallis test was performed by employing Dunn’s multiple comparisons to assess the significance of differences in abundances. Comparisons showing significant differences (marked as * for p ≤ 0.05, ** for p ≤ 0.005, *** for p ≤ 0.0005) were further tested by the Mann Whitney test.

|  | **Kruskal-Wallis test significance** | | | | **Mann-Whitney (P-Value)** | | | |
| --- | --- | --- | --- | --- | --- | --- | --- | --- |
|  | **Firmicutes** | **Actinobacteria** | **Bacteroidetes** | **Proteobacteria** | **Firmicutes** | **Actinobacteria** | **Bacteroidetes** | **Proteobacteria** |
| Ge vs Af | *** | *** | *** | *** | < 0.0001 | < 0.0001 | < 0.0001 | < 0.0001 |
| Ge vs Er | *** | * | *** | *** | < 0.0001 | < 0.0001 | < 0.0001 | < 0.0001 |
| Ge vs In | *** | No | *** | ** | < 0.0001 | ND | < 0.0001 | < 0.0001 |
| Ge vs Iq | *** | *** | *** | *** | < 0.0001 | < 0.0001 | < 0.0001 | < 0.0001 |
| Ge vs Sy | *** | *** | *** | *** | < 0.0001 | < 0.0001 | < 0.0001 | < 0.0001 |
| Ge vs Ot | ** | No | *** | * | < 0.0001 | ND | < 0.0001 | 0.0009 |
| Af vs Er | No | No | No | No | ND | ND | ND | ND |
| Af vs In | No | No | No | No | ND | ND | ND | ND |
| Af vs Iq | No | No | No | No | ND | ND | ND | ND |
| Af vs Sy | No | No | No | No | ND | ND | ND | ND |
| Af vs Ot | No | No | No | No | ND | ND | ND | ND |
| Er vs In | No | No | No | No | ND | ND | ND | ND |
| Er vs Iq | No | No | No | No | ND | ND | ND | ND |
| Er vs Sy | No | No | No | No | ND | ND | ND | ND |
| Er vs Ot | No | No | No | No | ND | ND | ND | ND |
| In vs Iq | No | No | No | No | ND | ND | ND | ND |
| In vs Sy | No | No | No | No | ND | ND | ND | ND |
| In vs Ot | No | No | No | No | ND | ND | ND | ND |
| Iq vs Sy | No | No | No | No | ND | ND | ND | ND |
| Iq vs Ot | No | No | No | No | ND | ND | ND | ND |
| Sy vs Ot | No | No | No | No | ND | ND | ND | ND |

Af: Afghanistan; Er: Eritrea; Ge: Germany; In: Iran; Iq: Iraq; ND: Not determined; No: Not significant; Ot: Others; Sy: Syria.

## Supplemental table 9: Summary of PERMANOVA results based on stool samples

Bonferroni corrected p values as determined by Permutational Multivariate Analysis of Variance (PERMANOVA) on Bray-Curtis (upper right triangle) and Jaccard distance (lower left triangle) matrices.

|  | **Germany** | **Syria** | **Iraq** | **Iran** | **Afghanistan** | **Eritrea** | **Others** |
| --- | --- | --- | --- | --- | --- | --- | --- |
| **Germany** | ^…^ | 0.00021 | 0.00021 | 0.00021 | 0.00021 | 0.00021 | 0.00021 |
| **Syria** | 0.00021 | ^…^ | 0.1275 | 0.9513 | 0.0063 | 0.1451 | 1 |
| **Iraq** | 0.00021 | 1 | ^…^ | 0.2898 | 1 | 1 | 1 |
| **Iran** | 0.00021 | 1 | 0.1144 | ^…^ | 0.0378 | 0.1323 | 1 |
| **Afghanistan** | 0.00021 | 0.00042 | 1 | 0.00966 | ^….^ | 1 | 1 |
| **Eritrea** | 0.00021 | 0.00042 | 0.00735 | 0.00399 | 0.05964 | ^….^ | 1 |
| **Others** | 0.00021 | 1 | 1 | 1 | 1 | 1 | ^….^ |

## Supplemental table 10: Prevalence of MDROs the refugee population in swab samples

|  | Prevalence in % (total number) | | | | | |
| --- | --- | --- | --- | --- | --- | --- |
|  | Germany^1)^ | all Refugees | Syria | Iraq | Afghanistan | other origins |
| MRSA | 0.7 - 1.3^IKMB^ | 6.3 (32/506) | 6.8 (16/235) | 9.6 (11/115) | 3.2 (3/95) | 3.3 (2/61) |
| ESBL | 0.0 - 6.3^3)^ | 1.6 (8/506) | 1.7 (4/235) | 2.6 (3/115) | 1.1 (1/95) | 0.0 (0/61) |
| CRE | 0.0 - 0.03^4)^ | 0.0 (0/506) | 0.0 (0/235) | 0.0 (0/115) | 0.0 (0/95) | 0.0 (0/61) |
| VRE | 0.0 - 1.0^5)^ | 0.0 (0/506) | 0.0 (0/235) | 0.0 (0/115) | 0.0 (0/95) | 0.0 (0/61) |

All data collected for refugees origins from oral, nasal and groin swabs.

1) Literature based prevalences for non-hospitalized German population: Köck R et al. 2015; Mehraj J et al. 2014; Belmar Campos et al. 2014; Valenza G et al. 2014; Lietzau S et al. 2005; Wendt C et al. 1999

IKMB Data origins from nasal swabs only

3) Data origins from rectal swabs and stool samples

4) Data origins from nasal swabs and stool samples

5) Data origins from rectal swabs and stool samples
